# Archetypes of bad physician leaders – a qualitative study from a large Finnish central hospital

**DOI:** 10.1108/LHS-12-2024-0154

**Published:** 2025-05-27

**Authors:** Sari Huikko-Tarvainen, Tommi Auvinen

**Affiliations:** Faculty of Medicine, University of Oulu, Oulu, Finland; School of Business and Economics, University of Jyväskylä, Jyvaskyla, Finland

**Keywords:** Physician leadership, Bad leader, Archetype, Health care

## Abstract

**Purpose:**

This study aims to explore the archetypes of bad leaders as perceived by Finnish physicians across various hierarchical levels. Understanding these archetypes is essential for addressing leadership deficiencies and mitigating the detrimental effects of bad leadership and the positivity bias in leadership research.

**Design/methodology/approach:**

Data were collected through in-person, semi-structured interviews with Finnish physicians (*n* = 50), including residents, specialists, heads of departments and chief physicians. Inductive content analysis, followed by thematization, was used to identify recurring themes and patterns of bad leadership behaviors. Initial codes were generated and subsequently categorized into archetypes of bad leadership, which were further organized into broader thematic categories of bad leadership.

**Findings:**

Based on our findings, the authors identified four main themes of bad leadership encompassing seven archetypes of bad leaders, addressing incompetence, lack of transparency, exploitation, bad workload management, bullying, psychological harassment and inequity: lack of support and accessibility (absent and dismissive), authoritarianism and abuse of power (autocratic, bully and manipulative), incompetence and ineffectiveness (incompetent) and unfairness and discrimination (unfair). These issues occurred occasionally in different situations.

**Practical implications:**

The findings indicate that a leader’s behavior and leadership style directly affect physicians’ well-being and work satisfaction, potentially contributing to higher physician turnover and reduced quality of patient care. These results underscore the importance of fostering leadership education that emphasizes communication skills, emotional intelligence and conflict resolution to enhance constructive leadership behaviors.

**Originality/value:**

To the best of the authors’ knowledge, no prior empirical research has specifically examined the archetypes of bad physician leaders.

## Introduction

Leadership in health-care settings is a fundamental determinant of both organizational performance and the well-being of health-care professionals (Clay-Williams *et al.*, 2017; Savage *et al.*, 2020). It also plays a pivotal role in shaping organizational culture, emphasizing the need to develop behaviors, strategies and qualities essential for effective leadership (West *et al.*, 2015). The role of physician leadership is unique due to the dual responsibilities of clinical expertise and administrative oversight (Prenestini *et al.*, 2021). Unlike nonclinical managers, physician leaders must navigate the complexities of patient care while ensuring the efficient operation of health-care organizations (Kirkpatrick *et al.*, 2021). Moreover, physician leadership is central to fostering a culture of high-quality patient care, facilitating team collaboration and supporting the personal and professional development of the medical workforce (Clay-Williams *et al.*, 2017; Kaiser *et al.*, 2020; Lyons *et al.*, 2020; Savage *et al.*, 2020). While leadership development programs can enhance organizational performance and improve patient outcomes (Lindgren and Gordon, 2016), physician leadership education is often inconsistently integrated into undergraduate medical curricula (Kellett *et al.*, 2015).

As with leadership research in general, physician leadership has focused more on good leadership. Good physician leadership is characterized by elements such as successful communication, trust, emotional intelligence, fairness, collegial respect, collaborative decision-making and the ability to support and develop others (Huikko-Tarvainen, 2022; Oskarsson and Vik, 2024). Previous research emphasizes the importance of supportive, transparent and competent leadership in creating a positive work environment (Hopkins *et al.*, 2015; Huikko-Tarvainen, 2022; van de Riet *et al.*, 2019). Conversely, bad leadership can manifest in various ways, all of which can severely undermine organizational effectiveness (Aasland *et al.*, 2010; Huikko-Tarvainen *et al.*, 2022). A recent review highlights the strong link between leadership styles and the quality of patient care, emphasizing the vital role of leadership in ensuring well-coordinated and integrated care delivery for both patients and health-care professionals (Sfantou *et al.*, 2017). Leadership in health care is also argued to be a “contextually influenced and context-producing practice” (Endrissat and von Arx, 2013). Failure to establish a quality workplace ultimately undermines patient care (Sfantou *et al.*, 2017). In addition, bad physician leadership can result in significant negative outcomes, including turnover (Huikko-Tarvainen *et al.*, 2022).

Moreover, physician leaders’ behavior is argued to significantly impact the well-being and burnout of the physicians they lead (Shanafelt and Noseworthy, 2017). Although leaders may exhibit a mix of constructive and destructive behaviors, indicating that leadership encompasses a range of actions rather than being purely positive or negative (Aasland *et al.*, 2010), little attention has been paid to specific archetypes of bad physician leadership and their contributions to organizational culture. Experiencing both constructive and destructive behaviors from the same leader can be more stressful than experiencing only destructive behavior (Duffy *et al.*, 2002). Furthermore, a destructive leader struggles to inspire trust and motivate followers (Schyns and Schilling, 2013), which is fundamental to remember given the global shortage of physicians (Butt *et al.*, 2024). Therefore, exploring the specific characteristics of bad physician leadership is vital for addressing these challenges and improving health-care system functioning.

## Theoretical framework

### Good and bad physician leadership

There is a limited but growing body of research on good and bad physician leadership (Huikko-Tarvainen, 2022; Huikko-Tarvainen *et al.*, 2022). Health-care leadership often aligns with transformational leadership, a form of constructive leadership (Schyns and Schilling, 2013), which highlights that supportive, motivational and emotionally intelligent leaders foster positive work environments and improve patient outcomes (Guevara *et al.*, 2019; Yukl, 2013; Yukl and Gardner, 2020). Transformational leadership is characterized by idealized influence, inspirational motivation, intellectual stimulation, individualized consideration and a focus on caring (Gabriel, 2015). Physician leaders who embody these traits serve as role models, inspiring, challenging and supporting their subordinates (Huikko-Tarvainen, 2022).

According to Gabriel (2015, 318), “The distinction between good and bad leaders reveals a fundamental experiential dichotomy that sets leaders apart from other individuals or groups, such as plumbers, therapists, and managers. They too can be described as good and bad, but such distinctions are based on their professional competence, skills, and abilities” (Gabriel, 2015). This resonates with professions with strong identities, such as engineers (Rottmann *et al.*, 2015) and, particularly, physicians (Huikko-Tarvainen *et al.*, 2021). Most studies on good leadership assert that leadership has a moral purpose, with leaders generally aiming for positive outcomes (Ciulla, 2005; Clements and Washbush, 1999). However, leaders may sometimes act unethically, publicly endorsing ideas they privately oppose or hiding information for personal gain (Bass, 1998). Thus, even leaders considered “good” may resort to manipulation in an effort to avoid autocracy or shield their followers from emotional burdens (Auvinen *et al.*, 2013).

Bad leadership manifests in harmful behaviors such as destructive, abusive, bossy, absentee, despotic, narcissistic, toxic, negative, petty tyranny, psychopathic, tyrannical, authoritarianism, incompetence, bullying, laissez-faire, supportive–disloyal, derailed, aversive, corrupt, evil, exploitative, insincere, insular, exclusion, incivility and undermining, which create an unhealthy work environment (Aasland *et al.*, 2010; Hogan *et al.*, 2021; Mackey *et al.*, 2021; Schyns and Schilling, 2013). These negative behaviors lead to decreased job satisfaction, higher stress levels, increased staff turnover and negatively impact commitment to work, employee well-being and individual performance. They also worsen attitudes toward leadership and satisfaction with leaders (Huikko-Tarvainen *et al.*, 2022; Krasikova *et al.*, 2013; Schyns and Schilling, 2013). In a Norwegian study, the prevalence of destructive leadership behavior was found to range from 33.5% to 61% among working-age employees (*n* = 2,539), indicating that destructive leadership is not an anomaly (Aasland *et al.*, 2010). In health care, although research on bad leadership is scarcer, behaviors such as micromanagement, emotional unavailability and public humiliation have been shown to foster a culture of fear and mistrust, ultimately harming both staff well-being and patient care (Huikko-Tarvainen *et al.*, 2022).

Leadership lacking empathy, communication and respect fosters a culture where physicians feel undervalued and overworked, with detrimental effects on their mental health (Maslach and Leiter, 2016). For example, physician burnout, a growing concern, has been shown to be exacerbated by bad leadership (Shanafelt and Noseworthy, 2017), as a clear link has been established between burnout and work overload, lack of control, insufficient recognition, lack of support and trust, unresolved conflicts and perceived injustice (Maslach and Leiter, 2016). Another key aspect of bad leadership in health care is equity and fairness. Leadership that perpetuates discrimination – whether based on gender, race or other factors – creates a toxic work environment that undermines morale. Studies have highlighted the persistence of gender disparities in health-care leadership, where female physicians often experience inequitable treatment in terms of pay, career advancement and workload distribution. (Carr *et al.*, 2018; Lydon *et al.*, 2022; Mousa *et al.*, 2021; Ringdahl *et al.*, 2014; Roth *et al.*, 2016.) In this study, bad leadership is defined as actions involving detrimental or unethical methods of influence that harm the organization or its employees or pursue goals conflicting with the organization’s legitimate interests.

The current study aims to deepen the understanding of how negative leadership behaviors recur and affect health-care professionals by exploring the lived experiences of physicians at various hierarchical levels. As Hogan *et al.* (2021) have noted, “The dark-side perspective helps correct a positivity bias in leadership research and provides insights for improving team, organizational, and national performance by minimizing the selection of the wrong people for leadership roles and enhancing the self-awareness and self-management of those in leadership roles” (Hogan *et al.*, 2021).

As actions, motivation and behavior can be influenced by perceptions (Koskiniemi *et al.*, 2019), these insights could provide a foundation for developing strategies to address leadership failures, improve physician leadership education and mitigate the harmful effects of bad leadership in health-care settings. Thus, “Professional integrity, as well as intellectual rigor, requires that we attempt to examine bad while we attempt to grow good” (Kellerman, 2024).

## Data and methods

The subjects of this empirical study were physicians of the Central Finland Health Care District, in which approximately 800 physicians are employed (Finnish Medical Association, 2016). The informants were invited to participate voluntarily in the study via an internal e-mail. All participants were eligible for inclusion in the study, as its objective was to identify elements of bad physician leadership as perceived by members of the doctor’s profession.

The data for this qualitative study were collected through semi-structured interviews with 50 Finnish physicians, representing various hierarchical levels, including 14 residents/specializing physicians, 13 specialists, eight heads of departments and 15 chief physicians working for a large Finnish central hospital. This composition provided a representative sample of the population of interest. To enhance the reliability of the study, interviews were conducted in person and in Finnish.

The interview instrument contained questions designed to explore perspectives on physician leadership from multiple angles, enabling participants to share their personal experiences with physician leadership. This approach aimed to elicit a diverse array of responses that were not restricted to a narrow or specific point of view, such as evaluating only, for example, physician leaders’ own work as leaders. Data specifically related to bad leadership were included in the present study. An English translation of the originally Finnish questionnaire is included in the Appendix.

To ensure the content validity of the study, the interview instrument was developed with care, informed by a review of the literature on bad physician leadership. Prior to full implementation, the instrument was pretested by the research team to evaluate the reliability and clarity of its components, as well as to identify any potential ambiguities or interpretive challenges. The feedback obtained during this pretesting phase helped to ensure that the instrument was both appropriate for the study population and easy to comprehend, while remaining consistent with the overall research objectives.

Inductive content analysis followed by thematization was used to examine the data. These methods are well-suited for exploring complex social phenomena. The analytical process comprised four steps (Elo and Kyngäs, 2008; Tuomi and Sarajärvi, 2018). The first step involved immersing oneself in the data by reading the interview transcripts multiple times to achieve a comprehensive understanding of the content. In the second step, initial codes were developed based on recurrent themes and significant statements related to bad physician leadership within the data. In the third step, similar codes were grouped into categories to identify patterns that reflected the experiences and perceptions of bad leadership among physicians. These categories were further analyzed to distill them into specific archetypes of bad leaders, capturing a range of detrimental behaviors and attitudes exhibited by physician leaders. In the fourth step, the identified archetypes were organized into broader themes that encapsulated the various aspects of bad physician leadership. This thematic analysis provided a structured understanding of the findings and illuminated the connections between different leadership failures. Illustrative excerpts from the interview data were carefully selected for each category in the results section.

This methodological rigor enhances the validity and reliability of the findings (Braun and Clarke, 2019). Furthermore, to strengthen the validity and reliability of the research findings, researcher triangulation was applied (Eriksson and Kovalainen, 2008) and the analysis was conducted twice and subsequently reviewed by both authors to reduce the potential for bias. As with any research instrument, limitations exist – including its restricted capacity to identify similarities due to an emphasis on differences. Thus, some overlap between different findings cannot be entirely ruled out.

The interviews were primarily conducted in person at the participants’ offices between April and June 2017 and between July and August 2018. All participants were informed about the purpose of the study and their right to withdraw at any time or refuse the use of their data. The identity of each informant was kept confidential and the interviews were coded in the excerpts (I1…I50). Consent for participation and data use were obtained from all participants, with data analysis conducted without personal identifiers. No incentives were provided for participation. The interviews were digitally recorded, transcribed and systematically coded for ease of analysis. Each interview lasted between 11 and 85 min, with a total duration of 25 h. The transcriptions produced 619 pages of A4 text (Calibri, 12-point font, single spacing). Data are available from the authors upon reasonable request.

### Ethical statement

This study follows national and international ethics standards for nonmedical research with human participants, adhering to guidelines set forth by the Finnish National Board on Research Integrity (TENK, 2019) and the data protection regulations of the European

Union (Finnish National Board on Research Integrity TENK guidelines 2019, 2019). Ethics approval was granted by the Central Finland Care District officials. This research did not receive any specific grant from funding agencies in the public, commercial or not-for-profit sectors.

## Results

In this section, the main themes of bad leadership and archetypes of bad leaders embedded within them will be discussed in more detail.

## Lack of support and accessibility

This theme includes absent leaders, who are characterized by physical or emotional absence, a lack of presence and unavailability when support is needed, and dismissive leaders, who show little concern for the well-being, contributions or feedback of their subordinates, often ignoring their needs and ideas. These results underscore the significance of leadership presence and emotional support in health care. Both absent and dismissive leaders contribute to a sense of isolation among physicians, particularly in challenging situations, which intensify physicians’ stress and dissatisfaction.

### Absent leader lacks presence and accessibility and, neglects emotional support

Several physicians expressed frustration with absent leaders who were physically distant and unavailable, especially during critical situations. Participants described their experience of lacking their leader’s presence in the following way:

I was working at a health center with a younger colleague, and the supervising doctor was at another center many kilometers away, available only by phone […] there was no real leadership presence […] I just felt like this is what working as a doctor is like, you’re doing it on your own without any leadership. There’s a nominal chief doctor at another health center, whom you see maybe once a month in a meeting, and they occasionally call to check how things are going […] Leadership should be present, it should be accessible. (I24).

When I personally experienced a patient's death, I just left work gritting my teeth, and there was no supervision offered. It was more like, “Well, better luck next time, things will go smoother next time”. (I24).

Such absence creates a sense of isolation, particularly for junior physicians. Even during extremely distressing situations, like patient deaths, the supervisor failed to provide adequate emotional or professional support. This emotional distance can severely affect the mental well-being of subordinates.

### Dismissive leader ignores concerns and downplays emotional distress

Dismissive leaders were often indifferent to the struggles of their team. Physicians expressed frustration with dismissive reactions to serious concerns in the following way:

When I had previously told my supervisor that I was struggling to cope, their response was, “Me neither.” That was the support I got. (I50).

I wanted to talk to my supervisor about a difficult situation in my career, but they completely shut down. He didn’t accuse me of anything, but he didn’t want to listen either […] He either couldn’t or didn’t want to support me when I needed it. (I39).

I encountered a situation for the first time where my patient committed suicide […] My immediate supervisor was on vacation, so I turned to the acting supervisor, a specialist doctor, for help with my distress and difficult emotions. His response was, “Well, I don’t know what to tell you, I’ve never had a patient commit suicide.” I felt like he was implying that he was such a good doctor that it wouldn’t happen to him […] I think that if your subordinate has a problem, you can't just brush it off with a “Teflon” response like, “I don’t know what to do, good luck, goodbye.” (I34).

When physicians sought support during a difficult situation, the leader ignored the request for support. The dismissive supervisors failed to provide both professional (career) and psychological (emotional) support during critical times. This resulted in a sense of being left alone with uncertainty and emotional burden.

### Authoritarianism and abuse of power

This theme includes leaders who are autocratic, bullying and manipulative. The theme captures the misuse of leaders’ authority, making decisions without input and treating subordinates with disrespect. Autocratic leaders make unilateral decisions without consultation, whereas bullying leaders rely on intimidation and public shaming to assert control. Manipulative leaders further compromise fairness by engaging in favoritism and nepotism.

### Autocratic leader is secretive in decision-making and has authoritarian behavior

Autocratic leaders exerted excessive control over decisions, and often bypassed consultation or communication, resulting in feelings of disenfranchisement among subordinates, as demonstrated in the following excerpts:

That decisions are made behind people's backs all the time, and nothing is communicated—suddenly, a decision comes out of the blue, stating that such a decision was made two weeks ago, and all of a sudden, there's just an email saying that this will now be done this way, without any form of discussion. (I36).

I had been working at one workplace for about two years, and my two-year contract was ending on August 31st. In May, I was informed that I couldn’t take any summer vacation […] Instead, I was told I could take vacation in September. It felt unfair […] I thought, this can’t be real, this can’t be how it works […] I think they even said they'd pay it out in cash, that I’d just keep working until the end of August and then get the vacation money. (I49).

As authoritarian leaders tend to impose decisions unilaterally, they foster a climate of fear or pressure, as seen when a physician’s contract details were changed without any discussion. Decisions made without input or transparency cause frustration and confusion among physicians. Physicians described situations where decisions were made “behind people’s backs” and announced with little notice or explanation.

### Bully leader executes humiliation, public shaming and psychological harassment

Some physicians reported being humiliated publicly. These supervisors exemplify bully leaders who use their leadership position to demean or intimidate others. Another physician described a leader who would put down those at the bottom of the medical hierarchy, illustrating how bullying behavior can permeate leadership dynamics. The following excerpts reveal experiences of inappropriate leader behavior:

I went to have a discussion with [the leader], but what ended up happening was that in front of everyone else, me, who brought up the problem, was criticized and humiliated. (I26).

Experiences of injustice […] for instance, after a tough night shift, harsh feedback might be given when reviewing the cases handled during the night. The exhausted on-call doctor is put against the wall and critiqued in front of the whole clinic. (I35).

I’ve personally been bullied by my supervisor […] the sole purpose seemed to be to force me out of my previous specialization. (I36).

If something was done behind their [leaders] back, they would lash out completely […] the worst part was how they could mercilessly put down those at the bottom of the medical hierarchy if they noticed that someone didn’t quite understand or know something […] some form of bullying […] It is essentially psychological harassment. (I37).

The bully leader engages in psychological harassment, using their authority to belittle, humiliate or harass subordinates. Inappropriate behavior by the leader has even taken place publicly, which inevitably influences the entire organization, impacting both psychological well-being and trust among organizational members.

### Manipulative leader uses favoritism in hiring and nepotism

Physicians highlighted leaders who manipulated job postings or appointments to ensure preferred candidates were hired. This undermined transparency and fairness. Another related issue was nepotism, where positions were created for the spouses of desired candidates. The following excerpts reveal experiences of manipulative behavior and nepotism:

I've always thought that if a position is open, it's advertised in the medical journal or on the employment office's website […] But it still sometimes works like this: the job posting is up on some notice board for two hours, and someone who’s already earmarked for the position is notified, they apply, and then the posting is taken down before anyone else can apply. Then, of course, the person who was lined up gets the job. (I29).

And then there's this peculiar thing, especially in hospitals: If they want that specialist doctor, and the spouse is a doctor too, so they’ll find a position for the spouse as well. I find it really strange that things can work like this […] It undermines credibility. (I29).

As such, manipulative leaders used power and influence to exploit situations for their benefit, often creating opportunities for favoritism, nepotism or unethical behavior. Furthermore, bully leaders engaged in psychological harassment, using their authority to belittle, humiliate or harass subordinates, whereas manipulative leaders further compromised fairness by engaging in favoritism and nepotism.

## Incompetence and ineffectiveness

This theme focuses on leaders who lack essential knowledge or shirk responsibilities. Incompetent leaders frustrate subordinates by failing to grasp key aspects of their roles or by avoiding difficult tasks. Such behavior demoralizes physicians and diminishes team effectiveness.

### Incompetent leader lacks knowledge and escapes responsibilities

Some leaders lacked essential knowledge to make informed decisions, which caused friction with subordinates. The following excerpts illustrate the experiences of incompetent leaders:

I had a pretty unpleasant experience myself. I went to present to the chief physician, as there were two of us [physicians] in the same position, and I knew the other person had a higher salary than I did. I went to discuss whether it would be possible for us to at least have the same base salary since we were doing the same job. I made an appointment, and when I raised the issue, he responded, “Well, the other physician has a PhD.” I replied that a PhD is not linked to the base salary, even though it gives a 6% bonus. He [the leader] then tried to explain other salary issues but clearly wasn’t aware of the difference between base and total salary. I corrected him, and he got upset, slammed his fist on the table, and told me to “get the hell out of here.” I was stunned […] After a few seconds, I said, “I guess this meeting is over,” and left the room. Later, he explained to others that he just lost his temper, but he never apologized. I think it would have been correct to apologize. (I15).

[The leader] covers up their own laziness by making work schedules and assigning themselves to places where there is no work on that particular day […] they fumble around to escape all responsibilities. (I27).

I’ve seen a lot of that kind of “slippery” leadership, where only the bare minimum leadership role is performed, just administrative decisions are made, and with little effort, while the focus is on other matters. (I35).

If the leader lacks the necessary knowledge, skills or capability to manage effectively, it leads to poor decision-making and frustration among staff. If the leaders shirk responsibilities and avoid taking on their full duties, the led physicians are left in charge.

## Unfairness and discrimination

Gender discrimination and disparities in pay and workload distribution are central to this theme. Unfair leaders foster a discriminatory environment, particularly for female physicians, as evidenced by gender-biased hiring practices and the undermining of women’s careers due to the potential impact of motherhood. Salary and workload inequalities were also emphasized.

### Unfair leader practices gender discrimination, bias, inequitable workloads and compensation

Female physicians reported discriminatory practices based on gender, particularly in hiring, promotions and maternity leave, as illustrated in the following excerpts:

There are many things tied to being a woman, like being belittled or referred to in a patronizing way […] In job interviews, they ask if you plan to have children […] It’s clear that you only get a permanent position after having kids. You can also see clearly that if I suggest something regarding a procedure or a patient, it’s only taken seriously when a male doctor suggests it. (I50).

A previous supervisor doctor who was responsible for training once said to me, “You're reaching the age when you’ll start having children, and then your specialization plans will get sidelined.” I thought, no way, my career can’t be derailed just because I’m a woman […] It felt extremely unfair to use my gender and potential motherhood as a reason. (I20).

Gender disparity still exists […] not only in terms of pay but also in terms of work discrimination, like with maternity leave. It still happens that employment contracts are terminated when maternity leave starts […] I’ve also been asked in an interview, “As a married woman, how do you plan to manage this job?” (I29).

There was this physician working at the health center who felt they had too much work. They told the boss at the time that they wanted to reconsider their job role because they felt overworked and stuck in a salary pit compared to others with similar roles. The boss offered a one euro raise and suggested they take on the maternity clinic as well. The person left after that. (I14).

For example, career plans were dismissed because of potential motherhood. In addition, unfair workload distribution and pay discrepancies were highlighted. As such, the unfair leader perpetuates inequities and unjust treatment within the workplace, including favoritism and gender discrimination.

Based on our analysis, we constructed four main themes of bad leadership, which contain seven archetypes of bad leaders to summarize our findings addressing incompetence, lack of transparency, exploitation, bad workload management, bullying, psychological harassment and inequity:

- lack of support and accessibility (absent and dismissive);
- authoritarianism and abuse of power (autocratic, bully and manipulative);
- incompetence and ineffectiveness (incompetent); and
- unfairness and discrimination (unfair) (Table 1).

**Table 1.** Summarizing of themes of bad physician leadership and archetypes of bad leaders

Themes of bad leadership	Archetype of bad leaders	Description
Lack of support and accessibility	Absent	Leaders are physically or emotionally unavailable, leading to a lack of guidance and emotional support for subordinates
	Dismissive	Leaders show little concern for the well-being, emotional needs or feedback of their team members, often brushing off or downplaying concerns
	Autocratic	Leaders make unilateral decisions without consultation or transparency, causing frustration and disempowerment among staff
Authoritarianism and abuse of power	Bully	Leaders engage in psychological harassment, including public shaming, belittling and humiliating subordinates
	Manipulative	Leaders manipulate situations for personal gain, including favoritism and nepotism in hiring practices
Incompetence and ineffectiveness	Incompetent	Leaders lack the knowledge or capability to perform their roles effectively, leading to frustration and poor decision-making
Unfairness and discrimination	Unfair	Leaders perpetuate inequities in the workplace, including gender discrimination, unfair workloads and pay disparities

**Source(s):** Authors’ own work

Hence, bad leadership may contribute to higher turnover rates among physicians, exacerbating the physician shortage and potentially leading to a decline in the quality of patient care. The following excerpt aptly summarizes the result of bad leadership:

A colleague voted with their feet and left, and I realized that if she was leaving, I wouldn't stay either. (I14).

## Discussion

Our study identified seven archetypes of leadership behavior: absent, dismissive, autocratic, bully, manipulative, incompetent and unfair. These archetypes were not universally present but occurred in varying situations and reflect four broader themes of bad leadership:

1. lack of support and accessibility;
2. authoritarianism and abuse of power;
3. incompetence and ineffectiveness; and
4. unfairness and discrimination.

Based on our findings, the leader’s behavior and leadership style affect the well-being and work satisfaction of individual physicians. As stated in the previous literature, leadership is shaped by contextual factors and cannot be understood as entirely positive or negative (Endrissat and von Arx, 2013). The findings of the present study also align with this contextual view of leadership, which involves a spectrum of actions ranging from constructive to destructive (Aasland *et al.*, 2010). The findings also align with existing literature on destructive leadership, where similar negative behaviors have been shown to harm organizational culture and diminish work quality and effectiveness (Aasland *et al.*, 2010; Hogan *et al.*, 2021; Schyns and Schilling, 2013). In the following sections, we discuss the findings of the current study in light of previous literature.

### Incompetence and lack of transparency

Leaders shirking responsibilities and failing to fully engage in their leadership roles left others to assume their duties, a phenomenon consistent with the concept of absentee leaders who hold leadership positions but contribute minimally to their role (Hogan *et al.*, 2021). Consequently, there is a lack of guidance and support for led physicians when leaders are physically unavailable. In addition, physicians expressed frustration over leaders’ inadequate understanding of clinical and administrative processes. This aligns with existing research emphasizing that physician leaders must possess both managerial and clinical expertise to lead effectively in health-care settings (Huikko-Tarvainen, 2022; Prenestini *et al.*, 2021). Incompetent leadership, as described by participants, also involved unilateral decision-making and a lack of consultation with frontline physicians, as supported by previous literature (Huikko-Tarvainen *et al.*, 2022). Some participants recounted decisions being made “behind people’s backs” (I36), reflecting a lack of transparency that fostered mistrust and dissatisfaction. Leaders who fail to engage their teams and make unilateral decisions create environments of disempowerment and disengagement, as evidenced by participants who described colleagues “voting with their feet” and leaving toxic work environments.

### Exploitation and workload management

Exploitation was another prevalent theme, with leaders assigning excessive workloads without regard for the capacity or well-being of their subordinates. Physicians reported being asked to take on additional responsibilities without adequate compensation or support. Excessive workloads and a lack of leadership support are significant drivers of burnout, which can lead to mental health issues (Maslach and Leiter, 2016). Factors influencing engagement and burnout, when misused among physicians, include workload and work demand, efficiency and available resources, a sense of purpose in their work, organizational culture and values, autonomy and flexibility, social support and community, as well as work–life balance (Shanafelt and Noseworthy, 2017). Notably, physician burnout not only increases turnover but also adversely affects patient care and satisfaction (Shanafelt and Noseworthy, 2017). Participants in this study described how concerns about workload were often dismissed or inadequately addressed by leadership, highlighting the exploitative nature of these behaviors and the importance of supportive leadership in mitigating burnout.

### Bullying and psychological harassment

Participants described instances of public humiliation and other behaviors intended to belittle or intimidate subordinates. These destructive leadership behaviors, such as bullying, are well-documented in the literature and are known to result in psychological harm, create hostile work environments, increase staff turnover and reduce job satisfaction and overall performance (Krasikova *et al.*, 2013; Schyns and Schilling, 2013). Bullying can also cause followers to feel a loss of control, intensifying stress levels (Schyns and Schilling, 2013). Furthermore, participants reported feeling demoralized and unsupported, with some leaving their positions due to bullying. These accounts highlight the detrimental impact of bullying on leadership in health care, especially given the global shortage of physicians (Butt *et al.*, 2024).

### Gender discrimination and inequity

Gender discrimination was also a noticeable issue, with female physicians reporting experiences of being belittled, discriminated against or passed over for opportunities based on their gender. These findings are consistent with literature documenting persistent gender disparities in the medical profession, particularly in leadership roles (Carr *et al.*, 2018; Lydon *et al.*, 2022; Mousa *et al.*, 2021; Ringdahl *et al.*, 2014; Roth *et al.*, 2016; Santucci *et al.*, 2023). Female participants expressed frustration over being judged based on their potential to have children or being patronized during interviews and evaluations (I20, I29). These issues underscore the importance of equity and inclusion in health-care leadership, particularly as more women worldwide enter the medical field (Finnish Medical Association, 2019). Leaders who perpetuate gender biases contribute to toxic work environments where female physicians feel undervalued and marginalized, resulting in decreased work satisfaction and increased turnover.

### Absence of emotional support and compassion

Some participants discussed the lack of emotional support from their leaders during times of personal or professional distress. This lack of empathy and compassion is a key characteristic of bad leadership, with leaders described as emotionally unavailable or indifferent (I39, I34). Emotional support and empathy are vital components of effective leadership, particularly in health care (Guevara *et al.*, 2019; Hopkins *et al.*, 2015), where employees frequently face high-stress situations (Shanafelt and Noseworthy, 2017) and where suicide rates among physicians are higher compared to those in the general population (Zimmermann *et al.*, 2024). Previous studies have also shown that physician leaders who demonstrate emotional intelligence and provide support during difficult times can significantly improve the morale and well-being of their teams (Östergård *et al.*, 2023; West *et al.*, 2015). Hence, leaders who fail to offer this support risk alienating their subordinates and contributing to a culture of emotional neglect. Participants in this study described feeling unsupported and even dismissed by their leaders when seeking help with difficult emotions, especially those related to patient deaths or challenging professional circumstances (I24). This lack of emotional availability further exacerbates the stress and burnout experienced by physicians, highlighting the need for leaders who are not only competent but also compassionate (Hopkins *et al.*, 2015). In conclusion, the findings of the current study underscore several far-reaching consequences of bad physician leadership.

## Strengths, limitations and future research

The semi-structured interviews enabled a thorough exploration of participants’ perspectives, yielding valuable insights into bad leadership. The diverse sample, including physicians from various hierarchical levels, ensured broad representation across the medical profession. Despite these strengths, several limitations remain. Inductive content analysis provided a structured framework for identifying and categorizing archetypes of bad leadership, enhancing the validity of the findings. However, reliance on self-reported data may introduce bias, as participants might underreport negative experiences due to fear of consequences. In addition, focusing solely on Finnish physicians limits the generalizability of the findings to other health-care professionals or cultural contexts (Onyura *et al.*, 2019). Future research should include cross-cultural comparative studies to assess the universality of these leadership archetypes. Furthermore, longitudinal studies could explore the long-term effects of bad leadership on physician well-being, turnover and patient care outcomes.

## Conclusion

This study has highlighted significant challenges related to bad physician leadership, as articulated by physicians across various hierarchical levels. The analysis identified seven archetypes of bad leaders (absent, dismissive, autocratic, bully, manipulative, incompetent and unfair leaders), categorized into four broader themes of bad leadership:

1. lack of support and accessibility;
2. authoritarianism and abuse of power;
3. incompetence and ineffectiveness; and
4. unfairness and discrimination.

These issues were occasionally present in different situations and encompassed incompetence, lack of transparency, exploitation, insufficient workload management, bullying, psychological harassment, gender discrimination and inequity. Based on the findings, a leader’s behavior and leadership style directly affect physicians’ well-being and work satisfaction, which can contribute to several far-reaching consequences. As leaders can exhibit both constructive and destructive behaviors (Aasland *et al.*, 2010), this research emphasizes the need for physician leadership training programs to enhance constructive behaviors and reduce destructive ones. By not only recognizing but also acknowledging how bad leadership manifests, organizations and individuals can more effectively establish and promote good leadership.

## Appendix

### Research questions

How would you describe a good physician leadership?

Does a leader of physicians need to be a physician her/himself?

### Additional research questions for leaders

What kind of leader are you?

What career path have you taken to your current position and what does your job entail?

How would you define/explain physician leadership to someone who is unfamiliar with the concept?

What key aspects would you highlight as the most important in physician leadership?

Is physician leadership different from other types of leadership?

What areas do you focus on in your own leadership work?

- Filling out the leadership role form (form below)

What aspects of your leadership do you find:

- Easy/rewarding/positive?
- Difficult/challenging/stressful?

How could physician leadership work be supported?

What are your thoughts on the current leadership training for physicians?

How do you feel about balancing your work and personal life?

Is there anything else you would like to share about physician leadership?
